# Tracking Age-Linked Antibiotic Resistance Patterns through Building-Level Wastewater Analysis

**DOI:** 10.1021/acsestwater.5c00349

**Published:** 2025-11-07

**Authors:** Anna Pico-Tomàs, Alejandro Sanchís, Cristina Mejías-Molina, Marc Comas-Cufí, José Luis Balcázar, Sílvia Bofill-Mas, Helena Torrell, Núria Canela, Carles M. Borrego, Lluís Corominas

**Affiliations:** † Catalan Institute for Water Research (ICRA-CERCA), Girona 17003, Spain; ‡ 196633 Universitat de Girona, Girona 17003, Spain; § Laboratori de Virus Contaminants de l’Aigua i d’Aliments, Departament de Genètica, Microbiologia i Estadística, 16724 Universitat de Barcelona, Barcelona 08028, Spain; ∥ Institut de Recerca de l’Aigua (IdRA), Universitat de Barcelona, Barcelona 08028, Spain; ⊥ Departament d’Informàtica, Matemàtica Aplicada I Estadística, Universitat de Girona, Girona 17003, Spain; # 303231 Eurecat, Centre Tecnològic de Catalunya, Centre for Omic Sciences (COS), Unit Universitat Rovira i Virgili-EURECAT, Unique Scientific and Technical Infrastructures (ICTS), Reus 43204, Spain; ∇ Grup d’Ecologia Microbiana Molecular, Institut d’Ecologia Aquàtica, Universitat de Girona, Girona 17003, Spain

**Keywords:** antimicrobial resistance (AMR), wastewater-based epidemiology (WBE), resistome, mobilome, age-groups, sewers, passive sampling

## Abstract

Antimicrobial resistance (AMR) is a global health challenge, and monitoring different demographic populations can improve our understanding of its spread and prevalence in urban settlements. This study applies building-level wastewater-based epidemiology (WBE) to analyze the resistome and mobilome of age-segregated populations from an elementary school (School), a university residence (UnivRes), and an elderly care facility (ElderlyRes) all located in Girona (Catalonia, Spain). Metagenomic analyses were subsequently conducted to investigate differences in bacterial communities, antibiotic resistance genes (ARGs), and mobile genetic elements (MGEs). The results revealed age-linked variations in the relative abundance and diversity of ARGs. The wastewater collected at the School exhibited the highest abundance of ARGs, while the ElderlyRes showed the highest diversity. Furthermore, sequences affiliated with bacterial pathogens were more prevalent in samples from both the School and the ElderlyRes, emphasizing potential public health implications. Among the 12 bacterial genera most strongly correlated with ARGs (Pearson *R* > 0.7), 11 were identified as members of the gut microbiota, underscoring their predominant role as reservoirs of resistance compared to bacteria of environmental origin. By integrating localized wastewater sampling with metagenomics, our study uncovers demographic-specific resistome patterns, delivering actionable evidence to strengthen AMR surveillance and intervention strategies in urban populations.

## Graphical abstract

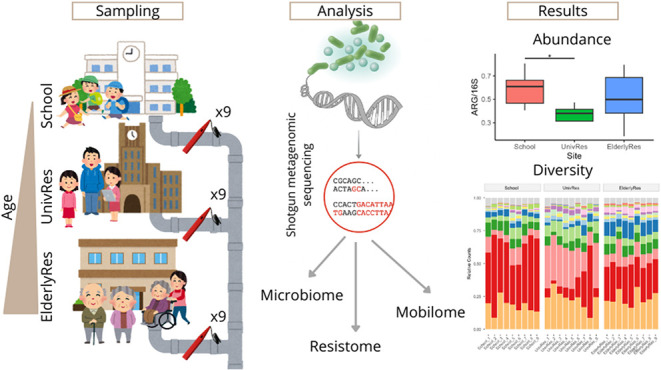

## 1 Introduction

The human gut serves as a reservoir for antimicrobial resistance genes (ARGs), collectively known as the resistome.^1^ Exposure to antibiotics either through therapeutic use or environmental sources exerts selective pressure on gut bacteria, fostering the proliferation and exchange of resistance genes. These resistant bacteria, along with their genetic material, are shed into wastewater systems, where they can disseminate further into the environment, contributing to the global antimicrobial resistance (AMR) burden.

Understanding how AMR varies across human populations is essential for developing targeted interventions to mitigate its spread. Age modulates a series of factors such as the immune system maturity, antibiotic usage patterns, dietary habits, and healthcare practices, which shape distinct gut microbiomes,^2–5^ and thus, different resistome profiles within communities.^6^ For instance, elderly individuals often exhibit a unique gut microbiota composition compared to children or young adults, influencing the abundance and diversity of resistant bacteria they host.^7^ Recognizing these population-specific differences is crucial for moving beyond one-size-fits-all approaches to AMR management, enabling more effective strategies such as age-specific antibiotic stewardship or tailored infection control measures.

Despite the variations in the carriage of antibiotic-resistant bacteria in the individual’s gut microbiota is well-known in relation to antibiotic usage,^8^ little is known about how the resistome varies across age groups or how these variations could inform AMR management strategies. Historically, research on AMR in humans has relied on clinical samples, predominantly blood^9^ or stool,^7,10,11^ collected from symptomatic patients with limited ability to capture population-wide trends. To overcome these limitations, wastewater-based epidemiology (WBE) has emerged as a powerful approach for monitoring AMR at the community level.^12–14^ WBE involves analyzing chemical or biological indicators in wastewater to extract health insights for entire populations. While most studies have focused on samples collected at the inlet of wastewater treatment plants (WWTPs), capturing signals from large and heterogeneous populations,^12–14^ finer-scale studies targeting specific buildings remain underexplored, despite the growing interest in monitoring wastewater from particular buildings (i.e., hospitals) during the SARS-CoV-2 pandemics.^15–17^ Here, we extend WBE to the building scale to investigate age related microbiome, resistome and mobilome patterns. We monitor communities representing distinct age groups: an elderly care residence (age over 65), a university dormitory (age ranging 17–25), and an elementary school (age ranging 3–12). Unlike previous studies relying on clinical or stool samples, this approach enables noninvasive, population-level monitoring of demographically homogeneous groups under real-world conditions. Specifically, we apply passive sampling, previously used for SARS-CoV-2 detection, now adapted for metagenomic analysis of the bacterial community, resistome and mobilome. Our findings aim to advance the understanding of age-linked AMR dynamics while serving as a proof-of-concept for applying WBE at the building level to monitor AMR in specific communities.

## 2 Materials and Methods

### 2.1 Study Sites

Three buildings in the city of Girona (Catalonia, Spain) were sampled between January and March 2022 (see Supporting Table S1 for their exact locations and sampling dates). These buildings house diverse age-grouped communities: (i) an elementary school (hereafter referred to as “School”) accommodating approximately 500 children aged 3–12 years, with adult teachers and staff (aged 25–60) comprising 10% of the population; (ii) a university residence (“UnivRes”) with 42 rooms, primarily occupied by young adults aged 17–25 years, with working staff representing about 4% of the residents; and (iii) an elderly residence (“ElderlyRes”) hosting 232 seniors over 65 years old, with staff workers constituting approximately 25% of the total population.

### 2.2 Wastewater Sampling

Sampling urban wastewater close to the source presents methodological challenges, primarily because the small size of sewer pipes limits the use of standard autosamplers, whose large dimensions make them unsuitable. We thus opted to use passive sampling devices (the so-called torpedoes) first described by Schang,^18^ and successfully applied to monitor SARS-CoV-2 in wastewater.^19^ The torpedo consists of a 3D-printed plastic case containing 3 cellulose ester membranes (EZ-Pak Membrane Filters, Millipore EZHAWG474) that is completely submerged in the wastewater for a defined period. During the time of exposure, wastewater flows inside the case, and the suspended solids (i.e., microorganisms, organic matter) are caught in the electronegative membranes. The use of torpedoes offers the advantage over grab sampling by providing time-integrated samples. The torpedoes were deployed for a period of 24 h, so each sample represented a day. Upon collection, samples were immediately transported to the laboratory at 0 °C in a portable icebox. Once there, the membranes were carefully removed from the plastic case, deposited individually in Eppendorf tubes and immediately stored at −20 °C until DNA extraction.

### 2.3 DNA Extraction

DNA was extracted from the collected filters using the FastDNA SPIN Kit for Soil (MP Biomedicals) in a final volume of 75 μL. The purity and concentration of DNA in each extract were quantified spectrophotometrically using NanoDrop (ThermoFisher Scientific) and fluorometrically using QUBIT 2 (Invitrogen), respectively. DNA extracts were stored at −20 °C until processing.

### 2.4 Metagenomic Sequencing

Sequencing was performed using Illumina chemistry. A total of 100 ng of the DNA was fragmented with the Bioruptor using 3 to 5 cycles of 30 s each, yielding fragments of approximately 350 bp. The fragmented DNA was used for library preparation with the TruSeq Nano DNA Library Prep Kit (Illumina). Library quality and quantity were assessed using Qubit and TapeStation. Libraries were pooled at equimolar concentrations and sequenced on the NextSeq 2000 System (150PE), generating at least 2 Gb per sample. Raw metagenomic sequences are published in the National Institutes of Health SRA database (https://www.ncbi.nlm.nih.gov/sra) under the accession number PRJNA1133228.

### 2.5 Data Analysis

Low-quality reads (less than 80% of bases with a Phred quality score >20) were removed using FASTX-toolkit^20^ to ensure the reliability of downstream analyses. ARGs were identified in the metagenomes using the DIAMOND^21^ algorithm against the ARGminer database^22^ (updated in 2021). In turn, MGEs were identified using an in-house constructed database of MGE indicators previously described by Gionchetta et al.^23^ METAXA software^24^ was used for the quantification of *16S rRNA* genes and their taxonomic assignment at the genus level. The composition of wastewater bacterial communities at the species level was analyzed following the pipeline in MEGAN6—Metagenome Analyzer,^25^ which includes the use of DIAMOND with the NCBI-nr database.^26^ Taxa associated with the human gut were determined following the list from the Unified Human Gastrointestinal Genome (UHGG).^27^

Statistical tests were run in R software^28^ using packages *readxl*,^29^ *dplyr*,^30^ *tidyr*,^31^ *coda.base*,^32^ *mixOmics* ^33^ and *stats*. All plots were created in R using the package *ggplot2*.^34^ Normality was tested with the Shapiro-Wilk test. ANOVA tests were conducted to assess statistical significance for the differences between groups. A Principal Components Analysis (PCA) with the clustered log-ratio transformations (CLR) was done to assess the distribution of the samples according to their microbiome (Genus level) and their resistome (ARGs) composition, and a K-means clustering algorithm was used to determine the sample grouping. Zeros were replaced using the posterior probabilities of a Dirichlet-multinomial distribution, with parameters estimated via maximum likelihood.^35^ For the correlation analysis, the logarithm of the ratio between the ARGs and the *16S rRNA* gene, as well as between each genus and the *16S rRNA* gene and MGEs and *16S rRNA* gene, was calculated before assessing the Pearson correlation to evaluate the strength and significance of the resulting associations (ARG-Genus and ARG-MGE). Resulting *p*-values were corrected using the False Discovery Rate (FDR) correction.^36^ The correlation network was plotted using Cytoscape.^37^

## 3 Results and Discussion

### 3.1 Composition of Sewage Bacterial Communities across Different Age Groups

The relative abundance of the different bacterial taxa in the samples was estimated using the *16S rRNA* gene as a proxy.^38^ When normalized by the total number of reads, the bacterial abundance increased in the three buildings according to the age group of the residents, being lower at the School and higher in the EderlyRes (Figure 1A). Differences in the relative concentration of this gene likely reflected a difference in the composition of bacterial communities in the sampled wastewater.^39^

**Figure 1:**
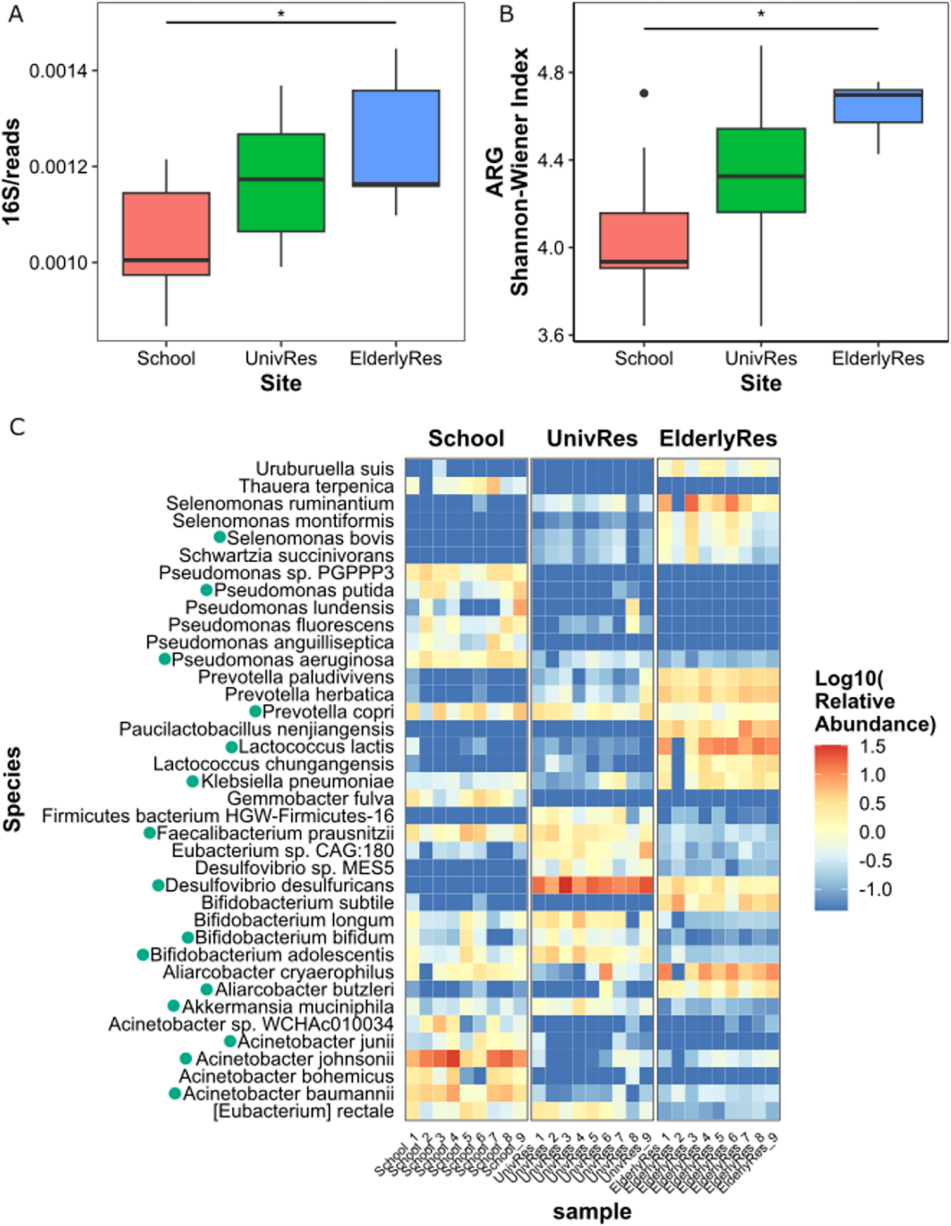
(A) Relative abundance of the *16S rRNA* gene (normalized by the total number of reads) in the three buildings. An ANOVA test showed statistically significant differences between the bacterial abundance in School and the ElderlyRes (*p*-value = 0.0158). (B) Bacterial diversity (i.e., Shannon–Wiener diversity index) of the wastewater bacterial communities in the three buildings. (C) Heatmap showing the 10 most abundant species in each sample. The color scale represents the normalized abundance of sequences assigned to each species (log10 scale). Species marked with a green dot are those specifically ascribed to the human gut according to the Unified Human Gastrointestinal Genome database (UHGG).

Phylum-level composition showed clear variability, suggesting structural differences in bacterial communities (Supporting Figure S1). The most abundant phyla were Firmicutes, Proteobacteria, Bacteroidetes, Actinobacteria, and Verrucomicrobia with varying proportions. Overall, 980 genera were identified, from which 189 belonged to human associated microbiota. K-means clustering reveals a perfect grouping of samples by sampling site using both full communities (Supporting Figure S2A, model explains 27.2% of the variance) and only the human-associated genera (Supporting Figure S2B, model explains 43.9% of the variance). At the species level, a total of 1,866 species were identified, and no significant differences were spotted regarding the Shannon–Wiener diversity index across the buildings (Figure 1B). The 10 most abundant species in each sample are shown in Figure 1C, and it is worth mentioning that these most abundant species did not fully overlap across buildings. This observation suggests equally diverse but distinct microbiome profiles. In the wastewater collected at the School, *Acinetobacter johnsonii* (relative abundance ranged from 0.02 to 0.28%) was the dominant species, while in the UnivRes the highest prevalence was associated with *Desulfovibrio desulfuricans* (0.07–0.33%). At the ElderlyRes, the predominant species were *Selenomonas ruminantium* (0.01–0.17%), *Lactococcus lactis* (0.02–0.13%), and *Aliarcobacter butzleri* (0.01–0.04%). Of these species, only *Selenomonas ruminantium* is not described as a common member of the human gut. Overall, the replicates showed consistent trends over time within each building, but these trends differed between buildings, indicating a stable bacterial community for each age group during the sampling period.

The differences in bacterial abundance and composition highlighted how potentially age-linked factors such as diet, antibiotic usage, and health status of the population affected wastewater microbiota. A relevant finding from this analysis is the presence of species classified as either pathogenic or opportunistic pathogens. *Acinetobacter baumannii*, *A. johnsonii* and *Pseudomonas aeruginosa* were relevant in the sewage collected at the School, while *Aliarcobacter butzleri* and *Klebsiella pneumoniae* were prevalent in the ElderlyRes. *Acinetobacter* species have frequently been reported in the gut microbiome of children, with their abundance often linked to recent antibiotic treatments.^40^ Particularly, *A. johnsonii* is a common species in the children’s gut microbiome, with higher abundance observed in children with autism spectrum disorder.^41^ Similarly, *A. baumannii* has been detected in fecal samples from children, especially those suffering from sepsis.^42,43^ Although there are no specific studies on the prevalence of *P. aeruginosa* in the gut microbiome of healthy children, it is known to be associated with respiratory infections that are common in children, thus suggesting a possible indirect contribution. For the ElderlyRes, *A. butzleri*, has been reported in the gut microbiome of healthy individuals,^44,45^ though not specifically in elderly subjects. On the other hand, *K. pneumoniae* is more abundant in elderly populations compared to younger ones.^46^ Although there is no direct relation between the presence of those species in wastewater and the prevalence of disease in the populations, their presence should be pointed out.

It is worth noting that some of the most prevalent species identified in the sewage from the three buildings are not typical of the human microbiota but rather associated with either environmental sources or animal origin. Only 223 of the species identified were normal components of the human gut microbiota. This represents 15.6% of the species in the ElderlyRes, 14.7% in the UnivRes and 12.6% in the School. These results agree well with those described by Becsei et al.^47^ in urban wastewater and confirm that the sewage microbiome is not solely composed of human bacterial commensals, but it also receives inputs from other sources (e.g., water used for cleaning purposes, stormwater conveying environmental bacteria, as well as litter that can end up in the sewer system).^48–50^ Sewers harbor three primary bacterial habitats: wastewater, sediment, and biofilms. Wastewater consists principally of human excreta flushed from toilets and drains. Sediment forms a semipersistent habitat where solids settle, creating a niche for bacterial colonization, although it is periodically disrupted by water flow. Finally, biofilms are composed of resilient bacterial communities attached to the pipe surfaces.^51^ Thus, while wastewater samples are presumed to represent primarily human-associated bacteria, it also include microbes resuspended from sewer sediments or detached from sewer biofilms.^51^ Some genera, such as *Arcobacter*, *Acinetobacter*, *Aeromonas, Pseudomonas*, as well as sulfate-reducing bacteria (*Desulfovibrio*) are not usually prevalent in the human gut, but they are in the sewer.^52,53^

### 3.2 Wastewater Resistome and Mobilome across Age-Groups

Figure 2 shows the relative abundance of ARGs and MGEs in the sewage collected in the three buildings. ARGs were measured in a range between 0.19–0.81 ARG/*16S rRNA* and MGEs between 0.32–2.21 MGE/*16S rRNA*. Both ARGs and MGEs were detected in lower abundance in the sewage from the UnivRes compared to the other buildings, suggesting that children and the elderly populations carried higher levels of ARGs and MGEs than young adults. The observed relative abundances of ARGs and MGEs are consistent with those previously reported in studies using similar methodologies, albeit with different sample types. For instance, Raza et al.^54^ reported values ranging from 0.43 to 3.5 copies of ARG/*16S rRNA* in the influent of a WWTP in Korea. Our samples fall at the lower end of this range, in fact, slightly below it. Regarding MGEs, the only study employing the same database as ours and is not using fresh water is that of Bertrans-Tubau et al.,^55^ who obtained 0.712 ± 0.2 copies of MGE/*16S rRNA*. Although this value is lower than ours, it was derived from treated water samples, where reduced concentrations are expected.

**Figure 2:**
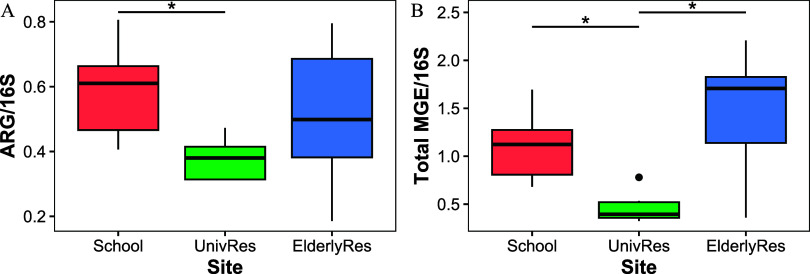
(A) Relative abundance of ARGs (reads identified as ARGs normalized by the total amount of *16S rRNA* genes) in the sewage from the three buildings sampled. (B) same for reads identified as MGEs. Asterisks denote statistical significance for the ANOVA test (ARGs: *p*-value = 0.0188; MGEs: *p*-value = 0.0000815).

A study by Fri et al.^6^ detected a larger amount of ARGs in the feces of young children compared to older children and adults. The authors attributed these differences to children’s less diverse, immature microbiota, which paradoxically harbored a greater abundance of ARGs. We observed differences in the ARG abundance between the populations of different age groups, while microbial diversity indexes remained similar. Another factor that can alter the abundance of ARGs is the consumption of antibiotics, which largely varies depending on age. Malo et al.^56^ determined that elderly adults (≥60 years) and children (0–9 years) were the age groups with the highest antibiotic consumption in comparison to other groups. Unfortunately, the lack of local antibiotic consumption data for the targeted populations precludes attributing the observed differences to variations in antibiotic prescription or usage, which is known to exert a selective pressure on the human microbiota, promoting the emergence and persistence of ARB.^2^ Despite this limitation, data from a national survey conducted in 2021 by the Spanish National Institute of Statistics indicate that the number of individuals aged over 65 years who had taken antibiotics in the previous 2 weeks was 3.2 times higher than among those aged 15–24 years (422,700 versus 133,200, respectively). This higher rate of antibiotic consumption in the elderly population is likely contributing to a greater burden of ARGs in their gut microbiota, which in turn, may help explain the increased prevalence of ARGs detected in wastewater collected from the elderly residence.

The diversity of ARGs increased according to the age group of the residents in each building, with statistically significant differences observed between the School and the ElderlyRes (Figure 3A). In contrast, no significant differences were found in the diversity indexes of MGEs across the different age groups (Figure 3B). Indeed, marker genes of plasmid (MOB genes), insertion sequences (ISCR/IS) and integron integrase genes (Int) were identified in all wastewater samples (Supporting Figure S3).

**Figure 3:**
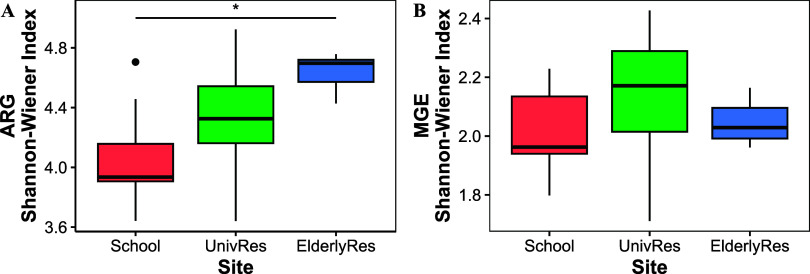
(A) Shannon–Wiener diversity index for the ARG in each building sampled. The asterisk denotes statistical significance between samples (ANOVA test, *p*-value = 0.00142). (B) Same as in A but for MGEs.

WBE poses challenges when interpreting diversity results, as observed differences may stem from two distinct but related factors. First, the higher diversity of ARGs measured in the ElderlyRes could reflect that each resident harbors a highly diverse community of ARB (and associated ARGs). This might be linked to cumulative antibiotic exposure throughout life,^11^ as elderly individuals are likely to have experienced repeated infections and periodic treatments with different antibiotics, which can shape their resistomes at the individual level. Second, greater diversity may also arise from differences between individuals within the population. In this case, the higher diversity in the wastewater from the ElderlyRes could be explained by the residents’ distinct clinical histories, shaped by diverse medical treatments and antibiotic consumption patterns. In contrast, children may share more homogeneous resistomes, likely influenced by similar healthcare exposures and treatments for common pediatric infections.^57^ These two factors (within-individual diversity and population-level differences) may interact, resulting in the observed patterns of ARG diversity.

Across the dataset, 902 different ARGs were identified. Figure 4 shows the abundance of the top 10 ARGs for each sample, grouped by the antibiotic families they confer resistance to. A clear distinction can be observed between the buildings, with sample replicates within each building showing similar patterns. A K-means clustering analysis including all the identified ARGs showed perfect clustering of the samples by site (Supporting Figure S4). In samples collected at the School and the ElderlyRes, multidrug resistance genes such as *mdtB*, *mdtC*, and *MexB* were abundant, indicating a higher prevalence of genes conferring resistance to different antibiotic classes. Conversely, genes conferring resistance to tetracyclines such as *tetW*, *tetQ*, and *tetO* were prevalent in the samples collected at the university residence. We also observed differences in the most abundant genes within the same family. For instance, in the Macrolide-Lincosamide-Streptogramin (MLS) family, the most abundant gene in the School sewage was *msrE*, while *ermB* was prevalent in wastewater from the elderly residence. Among the 10 most abundant genes, representatives from 7 out of 20 antibiotic families were present. Notably, genes conferring resistance to aminoglycosides and fluoroquinolones were absent from this top 10 list, despite these families ranking among the 7 most prevalent ARG families across all buildings when considering the total gene count (Supporting Figure S5). This absence is probably due to the uniform distribution of all the identified genes conferring resistance to these antibiotics, with no single gene dominating in abundance. Overall, the most prevalent ARGs were those conferring resistance to MLS, multidrug, tetracyclines, β-lactams, aminoglycosides, and fluoroquinolones. This pattern aligns with previous studies^58^ and with commonly dispensed antibiotic classes. National primary healthcare prescribing data from 2020 confirm that fluoroquinolones, macrolides (within MLS), and penicillins (within β-lactams) were among the most frequently prescribed antibiotics.^59^ This data supports our findings of prominent resistance genes against fluoroquinolones and β-lactams.

**Figure 4:**
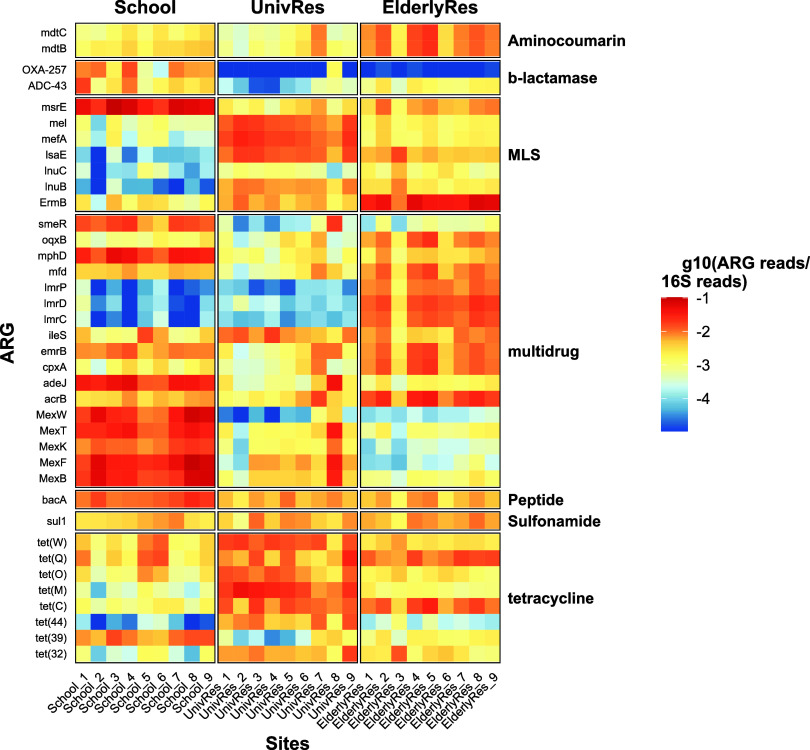
Heatmap of the relative abundance of ARGs (ARG copies/ *16S rRNA* gene copies) in the wastewater from each building.

Genes such as *msrE*, *mel*, *MexK*, *adeJ*, and *sul1* were found in high abundance, consistent with previous studies identifying them as dominant in global sewage samples. Conversely, other genes frequently reported in the literature, such as *ANT­(3*″*)-IIa_clust*, *bla*_OXA‑256_ *_clust*, *mphE*, *macB*, *mdtB*, and *qacH*, were present but not dominant in any sample. Notably, in some cases, variants of the expected genes were more prevalent, such as *bla*_OXA‑257_.^58^

Antibiotic treatment reduces bacterial diversity in the gut and increases the abundance of ARGs and plasmids, especially of core ARGs.^7^ The variation in ARGs conferring resistance against different antibiotics between buildings likely reflects differences in antibiotic exposure and consumption patterns among these populations. For example, the higher prevalence of multidrug resistance genes in the School and the ElderlyRes samples may be associated with increased exposure to multiple antibiotics among children and the elderly, potentially due to frequent infections and prophylactic antibiotic treatments. In contrast, the prevalence of genes encoding resistance to tetracyclines in the UnivRes samples may reflect distinct usage patterns of antibiotics among young adults. Tetracyclines are commonly used as treatments for severe acne (often affecting young adults) and sexually transmitted infections such as gonorrhea, syphilis, and chlamydiosis.^60,61^ Although we detected sequences affiliated with the genus *Treponema* in samples from UnivRes and ElderlyRes, this does not necessarily indicate active syphilis infections, as *Treponema* is a usual member of the normal gut microbiota.^62^ *N. gonorrheae* was not detected in any sample, whereas sequences affiliated with *Chlamydia* were only detected in a single sample from the School and three samples from the ElderlyRes. Human sample-based studies have determined that the main contributor to the resistome is the bacterial community, followed by antibiotic use, demographic variables (gender and socioeconomic status), geography, population density, and diet.^63^ Unfortunately, the lack of age-disaggregated antibiotic consumption data for our target populations precludes such type of correlations in our study.

The associated risk of the identified ARGs was defined according to Shuai et al.^64^ ranking system, which evaluates the risk based on three principal factors: the global abundance of the ARG in different environments, their mobility potential, and the host pathogenicity (Supporting Figure S6). Among the sampled buildings, the sewage collected at the UnivRes showed the highest proportion of ARGs classified as Rank I, followed by the School and the ElderlyRes. However, it is important to contextualize this finding. While the UnivRes exhibits a higher proportion of Rank I ARGs, it also has the lowest overall ARG abundance, highlighting the bias caused by analyzing only relative metrics. Furthermore, identifying a site as a potential “threat” based on its resistome profile should be approached cautiously, particularly when comparing buildings such as schools or residences to healthcare settings such as hospitals, where the abundance of pathogens and clinically relevant ARGs are prominent.

### 3.3 Correlation between Wastewater Bacteria, Mobile Genetic Elements and ARGs

A correlation analysis was performed to identify relations between bacterial taxa and ARGs. The resulting network is composed of a total of 70 nodes and 361 edges (significative interactions after FDR correction) and it consists in a single component as it is fully connected with a moderate density of 0.163. The average number of neighbors was 11.2, which indicates the average number of ARGs connected to each genus. The heterogeneity (1.05) and the centralization (0.67) coefficients indicate that few genera act as hubs and are linked to multiple ARGs, while most nodes have fewer connections. Figure 5 shows the strongest significant correlations (*R*_Pearson_ correlation coefficient >0.7) between the bacterial genera and ARGs with the *R*_Pearson_ values compiled in Supporting Figure S7. The correlation network shows five main clusters: the first one includes the genus *Bifidobacterium*, the second one includes *Desulfovibrio*, *Desulfitobacterium*, and *Acidaminococcus*; the third one includes *Enterobacter*, *Klebsiella*, *Veillonella*, and *Lactococcus*; the fourth includes *Pseudomonas*, *Acinetobacter*, and *Rheinheimera*; and the last one only includes *Mycobacterium*. To further investigate whether the genera grouped within each cluster also co-occur with one another, we calculated a Genus–Genus correlation matrix. This second analysis presented in Supporting Figure S8 revealed significant positive associations between the genera within each cluster.

**Figure 5:**
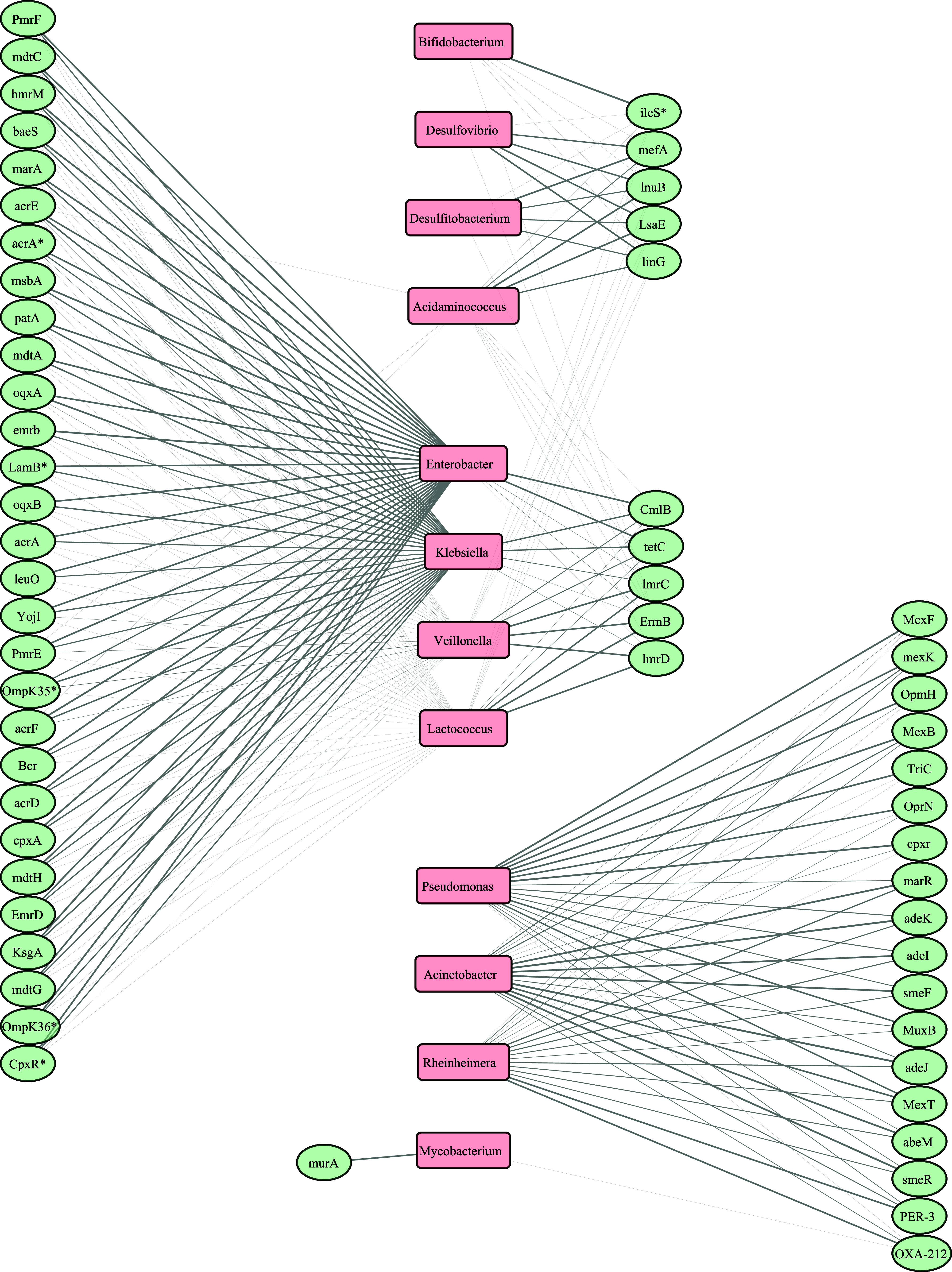
Correlation network between ARGs (green circles) and genera (pink rectangles). Connections represent positive correlations, with an *R*_Pearson_ > 0.7 and the width indicating the magnitude of the correlation. Asterisks denote gene abbreviations. Therefore, CpxR* stands for *Pseudomonas aeruginosa CpxR*, ileS* for *Bifidobacteria* intrinsic *ileS* conferring resistance to mupirocin, OmpK35* and OmpK36* for *K. pneumoniae* *OmpK35* and 36 respectively, LamB* for *Escherichia coli LamB* and acrA* for *Enterobacter cloacae acrA*.

Some ARGs that strongly correlated with these genera are also found among the most abundant, such as *ileS* (strongly correlated with *Bifidobacterium*, *R*_Pearson_ = 0.99), and *mefA*, *lnuB*, and *LsaE* (strongly correlated with *Acidaminococcus*, *Desulfovibrio* and *Desulfitobacterium*). From the second cluster, we observed, that *lmrC, lmrD and ermB* were strongly correlated with *Veillonella* and *Lactococcus, tetC* with *Enterobacter*, *Klebsiella*, and *Veillonella* And genes *mdtC*, *emrB*, *cpxA* correlated with *Enterobacter* and *Klebsiella*. For the third cluster, stronger associations were between genes *SmeR* and *adeJ* with *Acinetobacter*, and *MexT*, *MexK*, *MexF* and *MexB* with *Pseudomonas*. Lastly, a strong correlation was observed between *Mycobacterium* and *murA*. The ARGs and the species associated with genera *Acinetobacter* and *Pseudomonas* were highly prevalent at the School, *Lactococcus* was prevalent at the ElderlyRes, and *Desulfovibrio* at the UnivRes.

Of the 12 top genera correlated with ARGs, 11 belonged to the gut microbiota (all except *Rheinheimera*), suggesting that these genera are more prone to harbor ARGs than environmental bacteria. In some cases, biological relations exist between the genus and its most correlated ARGs, like for Bifidobacteria intrinsic *ileS* conferring resistance to mupirocin or *murA*, both related to the genus where these ARGs were first described.^65,66^ The same happens with the ARGs associated with *Pseudomonas*. In other cases, however, such a relationship has not been described yet, suggesting that the observed co-occurrence is probably related to the codistribution of the identified bacterial species with an unknown resistant strain. This might be the case for *Enterobacter*, which strongly correlates with ARGs that have already been described in *E. coli*. The correlation between the genus *Enterobacter* and *Escherichia*/*Shigella* is 0.82 (data not shown), suggesting proportional variation of these genera among samples, producing similar results when correlating them with the ARGs.

We also explored the associations between ARGs and MGEs (Supporting Figures S9 and S10). The resulting correlation network consisted of 65 nodes (50 ARGs and 15 MGEs) and 488 significant connections (after FDR correction) with a density of 0.26, indicating that about a quarter of all possible associations were present. Each node (MGE) was connected to an average of 16.5 ARG neighbors, with a heterogeneity of 0.66 and a centralization of 0.41, suggesting a relatively balanced distribution with no single node dominating the network. As a bipartite system, the clustering coefficient was 0. The most connected ARG was *mfd*, linked to 13 MGEs, while the most connected MGE was from the IS1380 family, associated with 38 ARGs. The strongest positive correlations primarily involved IS-family elements (IS3, IS21, and IS1380) and multidrug efflux genes (*adeJ*, *adeK*, *smeR*, or *mexW*). These strong correlations may indicate that these MGEs could facilitate the mobilization of efflux-related resistance determinants. Similar associations between IS elements and efflux pumps have been reported in environmental metagenomes and clinical isolates.^54,67^ However, these findings contradict Nielsen,^68^ who reported that efflux genes are rarely associated with insertion sequences. This discrepancy might be due to the fact that, although efflux genes do not appear within MGE contexts in reference genomes, they can co-occur in environmental samples, and therefore, potentially promote IS-mediated mobilization of these ARGs.

## 4 Conclusions

Our study reveals significant age-linked variations in the resistome and mobilome of wastewater samples collected from buildings. These results are comparable to what is observed in clinical settings, offering a noninvasive and population-wide approach to monitor age-associated antimicrobial resistance dynamics through wastewater, and demonstrating the potential of building-level WBE to complement clinical surveillance systems. The elderly population harbors the highest diversity of ARGs and MGEs, while children exhibit the highest ARG abundance. Metagenomic analysis provided a comprehensive overview of ARG and MGE profiles, pathogenic bacteria, and environmental inputs in the sampled sewers, underscoring the importance of demographic factors in AMR surveillance. Besides, the highest correlations between ARG and bacteria were found for human-derived rather than for environmental bacteria. However, challenges in distinguishing human-derived signals from sewer contributions highlight the need for methodological refinement. Future research should focus on expanding sampling to diverse settings and integrating clinical and antibiotic prescription and consumption data to enhance the utility of WBE for localized AMR monitoring to guide public health interventions.

## Supplementary Material



## Notes

Further details of the samples, ARG, MGE, and phylum profiles; K-means clustering charts; ARG rank classifications; and correlation matrices (PDF)
