# Supplementary material for: Tracking Age-Linked Antibiotic Resistance Patterns through Building-Level Wastewater Analysis
Source: ACS ES T Water. 2025 Nov 7;5(12):7141–51. doi: 10.1021/acsestwater.5c00349 (PMC12707230; doi:10.1021/acsestwater.5c00349)
Supplement: Supplementary file 1 [file ew5c00349_si_001.pdf]

Tracking Age-Linked Antibiotic Resistance Patterns Through Building-Level Wastewater Analysis

Authors:

Anna Pico-Tomàs[1 2*], Alejandro Sanchís[1], Cristina Mejías-Molina[3 4], Marc Comas-Cufí[5], José Luis Balcázar[1], Sílvia Bofill-Mas[3 4], Helena Torrell[6], Núria Canela[6], Carles M Borrego[1 7], Lluís Corominas[1]

[1] Catalan Institute for Water Research (ICRA-CERCA), Girona, 17003, Spain

[2] Universitat de Girona, Girona, 17003, Spain

[3] Laboratori de Virus Contaminants de l'Aigua i d'Aliments, Departament de Genètica, Microbiologia i Estadística, Universitat de Barcelona, Barcelona, 08028, Spain

[4] Institut de Recerca de l'Aigua (IdRA), Universitat de Barcelona, Barcelona, 08028, Spain

[5] Departament d'Informàtica, Matemàtica Aplicada I Estadística, Universitat de Girona, Girona, 17003, Spain

[6] Eurecat, Centre Tecnològic de Catalunya, Centre for Omic Sciences (COS), Joint Unit Universitat Rovira i Virgili-EURECAT, Unique Scientific and Technical Infrastructures (ICTS), Reus, 43204, Spain

[7] Grup d'Ecologia Microbiana Molecular, Institut d'Ecologia Aquàtica, Universitat de Girona, Girona, 17003, Spain

* Email: apico@icra.cat

Supplementary information

Supplementary Table S1. List of samples collected.

Sample	Site (coordinates)	Initial date	Final date
UnivRes_1	UnivRes (41.961760, 2.825300)	07/01/2022	08/01/2022
UnivRes_2		17/01/2022	18/01/2022
UnivRes_3		24/01/2022	25/01/2022
UnivRes_4		31/01/2022	01/02/2022
UnivRes_5		14/02/2022	15/02/2022
UnivRes_6		23/02/2022	24/02/2022
UnivRes_7		28/02/2022	01/03/2022
UnivRes_8		02/03/2022	03/03/2022
UnivRes_9		07/03/2022	08/03/2022
School_1	School (41.972996, 2.829481)	07/01/2022	08/01/2022
School_2		17/01/2022	18/01/2022
School_3		19/01/2022	20/01/2022
School_4		31/01/2022	01/02/2022
School_5		14/02/2022	15/02/2022
School_6		21/02/2022	22/02/2022
School_7		28/02/2022	01/03/2022
School_8		02/03/2022	03/03/2022
School_9		07/03/2022	08/03/2022
ElderlyRes_1	ElderlyRes (41.958438, 2.824287)	07/01/2022	08/01/2022
ElderlyRes_2		17/01/2022	18/01/2022
ElderlyRes_3		19/01/2022	20/01/2022
ElderlyRes_4		31/01/2022	01/02/2022
ElderlyRes_5		14/02/2022	15/02/2022
ElderlyRes_6		23/02/2022	24/02/2022
ElderlyRes_7		28/02/2022	01/03/2022
ElderlyRes_8		02/03/2022	03/03/2022
ElderlyRes_9		07/03/2022	08/03/2022

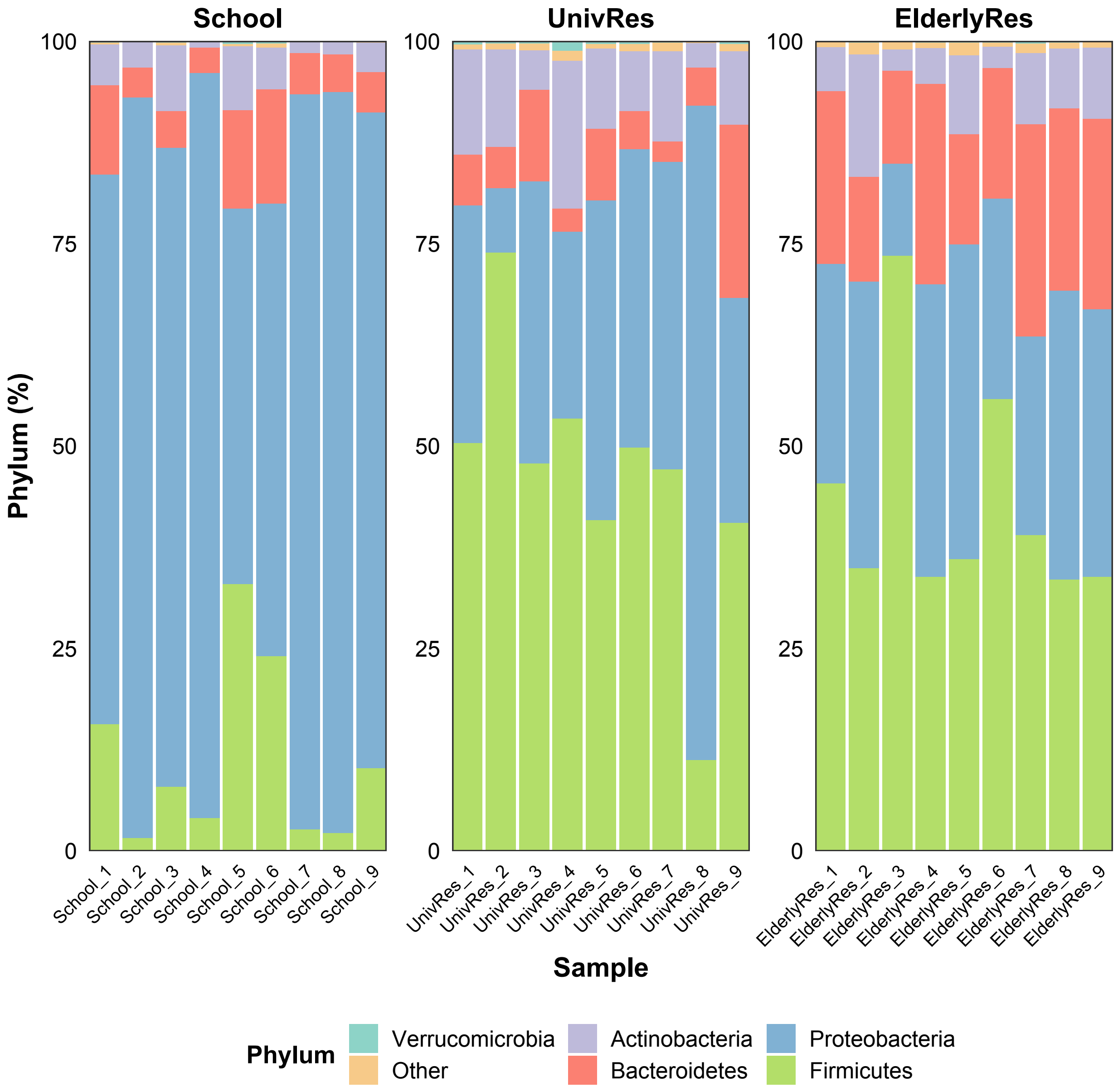

Supplementary Figure S1. Relative abundance of the different bacteria phyla in each sample. Only the 5 most abundant (>0.5%) are represented individually, the rest are grouped in Other.

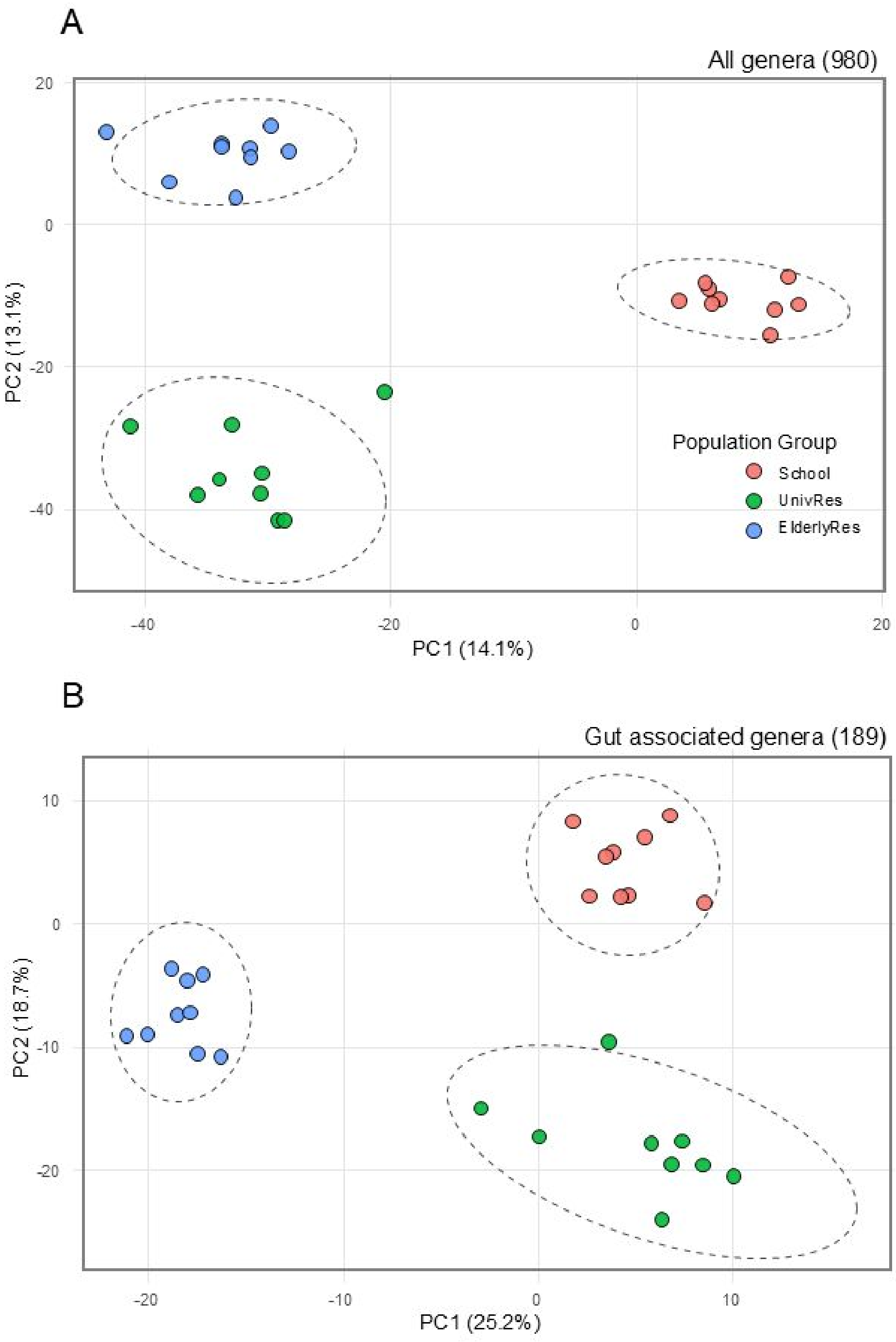

Supplementary Figure S2. PCA with K-means clustering of the microbial communities of the samples at the genus level. Fig. A uses all genera, whereas Fig. B uses only those genera

associated with the human gut as described in the Unified Human Gastrointestinal Genome database (UHGG).

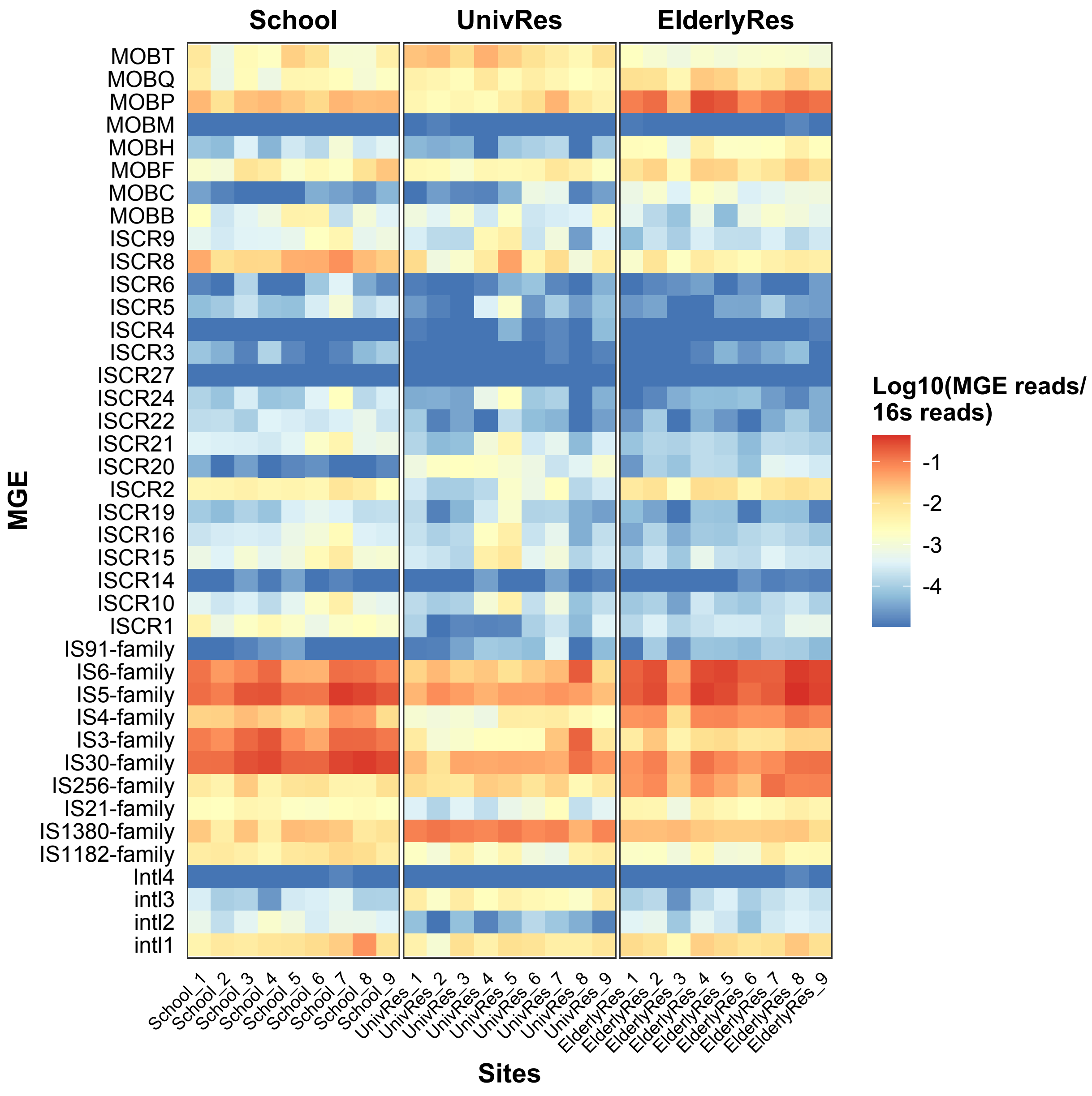

Supplementary Figure S3. Normalized abundance (MGEs reads/*16S rRNA* gene reads) of the identified MGE indicators.

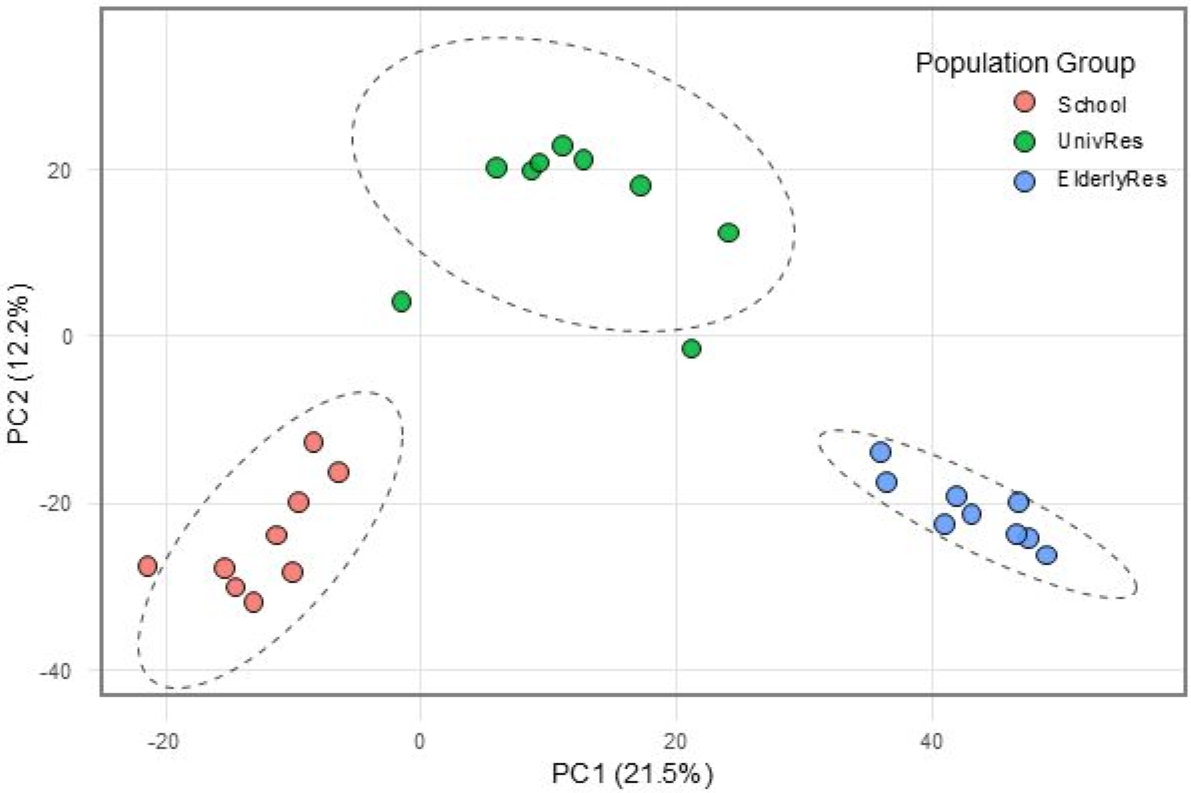

Supplementary Figure S4. PCA with K-means clustering of the resistome of each sample.

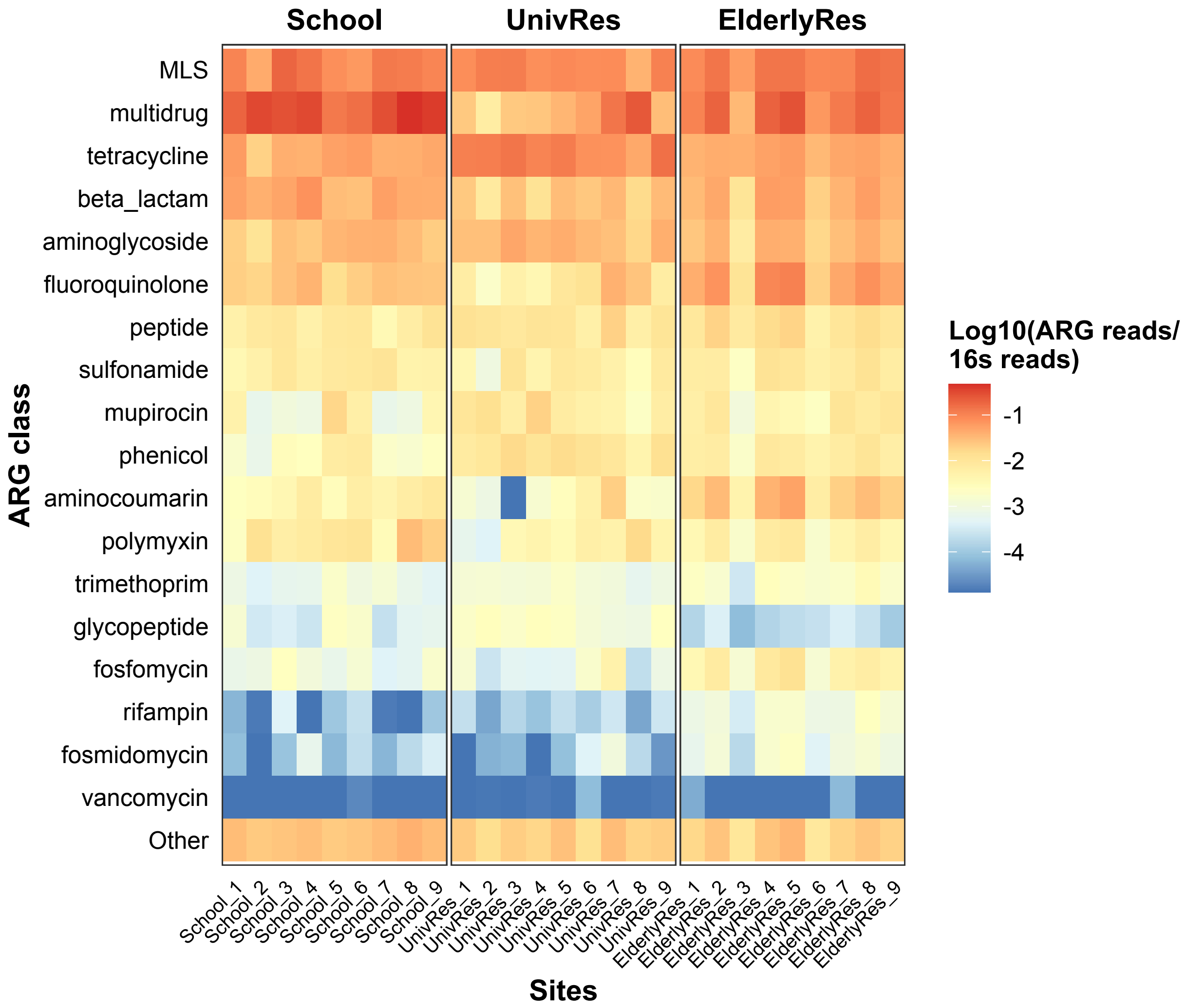

Supplementary Figure S5. Normalized abundance (ARGs reads/*16S rRNA* gene reads) of the identified ARGs grouped by the antibiotic family which they confer resistance to.

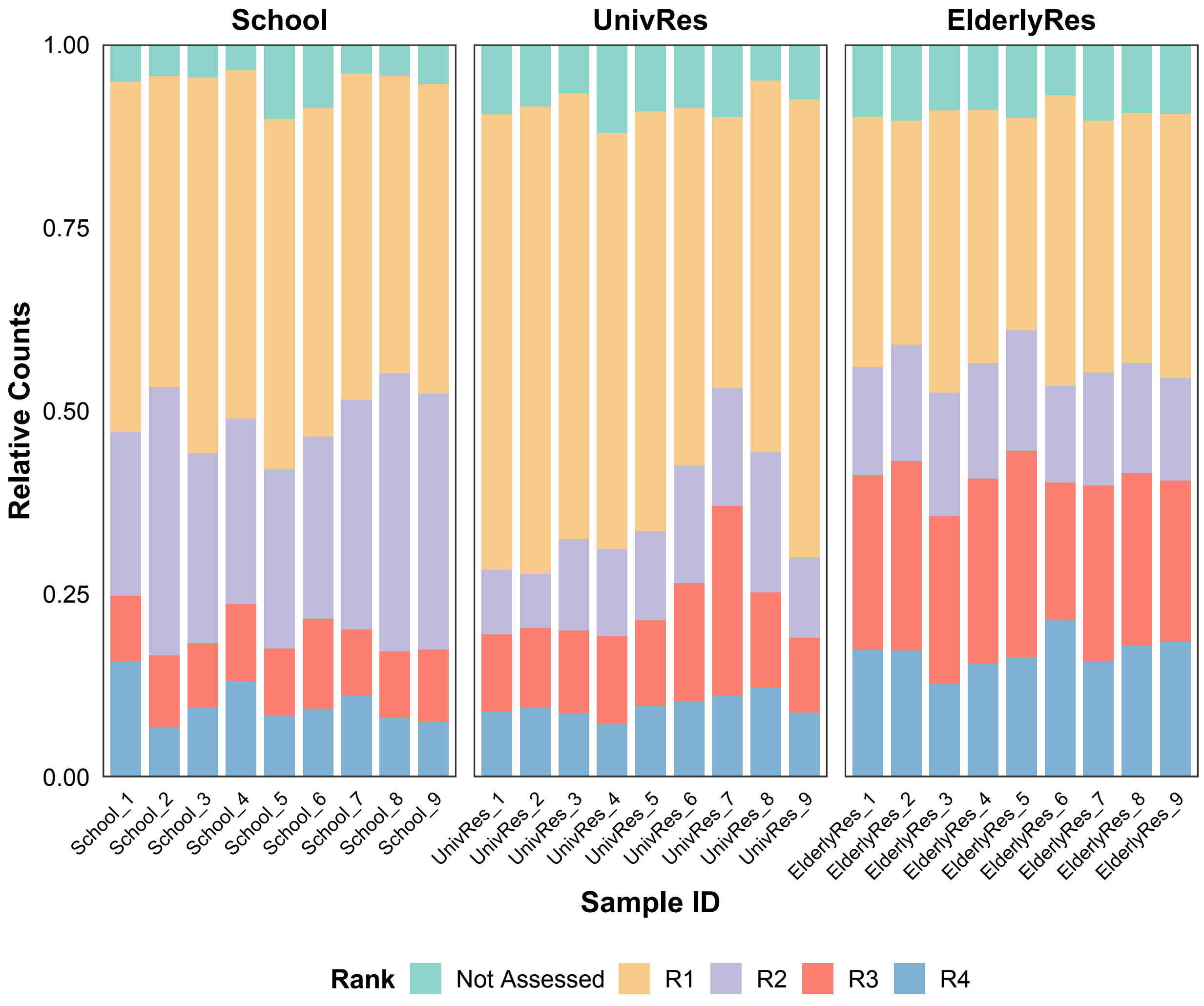

Supplementary Figure S6. Relative abundance of the of ARG grouped by priority ranks (defined by Shuang et al., 2024). R1 represents the highest risk while R4 represents the lowest. Not Assessed is for the genes that were assessed in the study, but no rank was given to them and for the genes that were not assessed in the study.

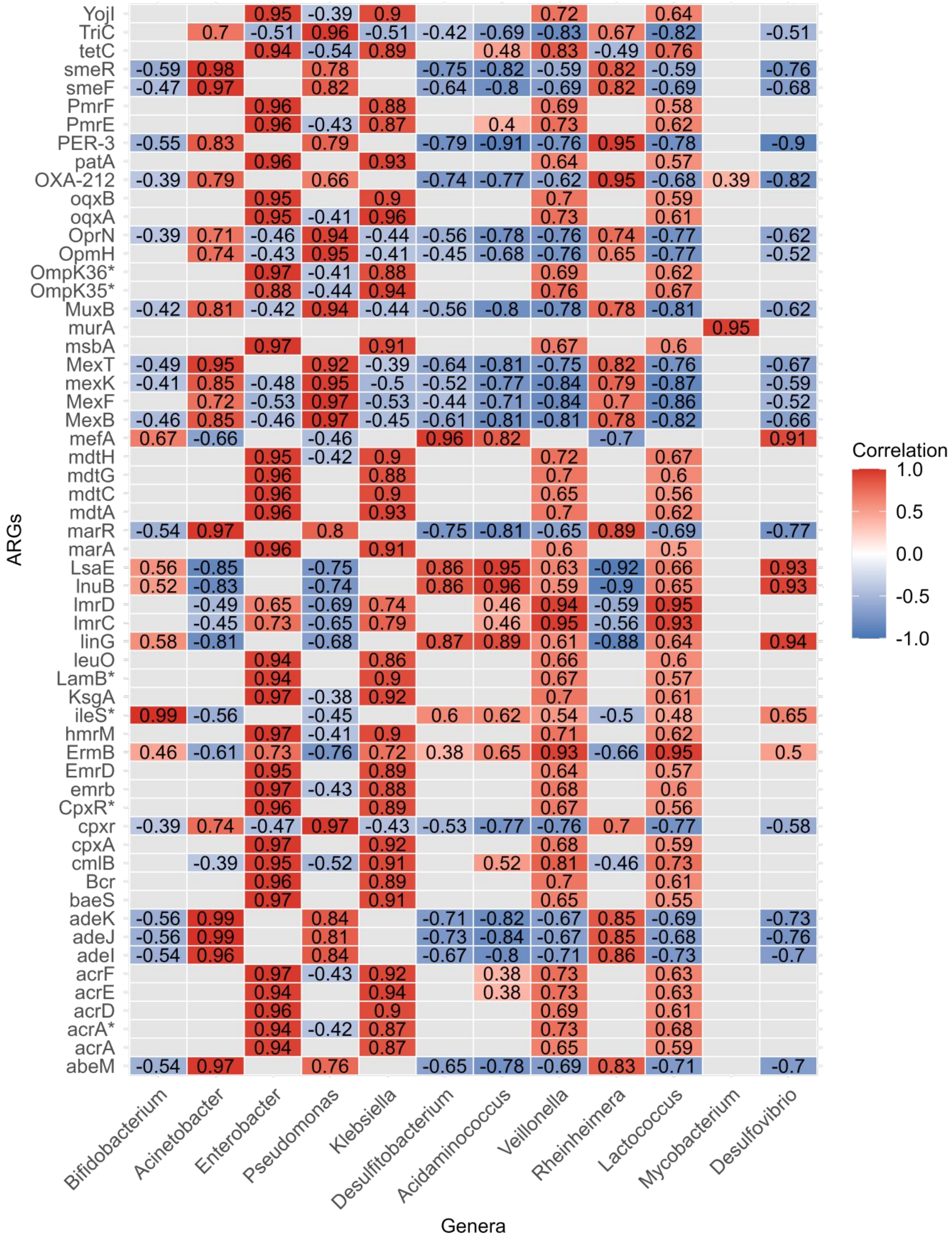

Supplementary Figure S7. Correlation matrix between ARGs and bacterial genera. The 30 strongest correlations were selected for representation and for the intersections only the significant correlations (after FDR correction) were colored. Numbers within cells correspond to the corresponding Pearson correlation coefficients.

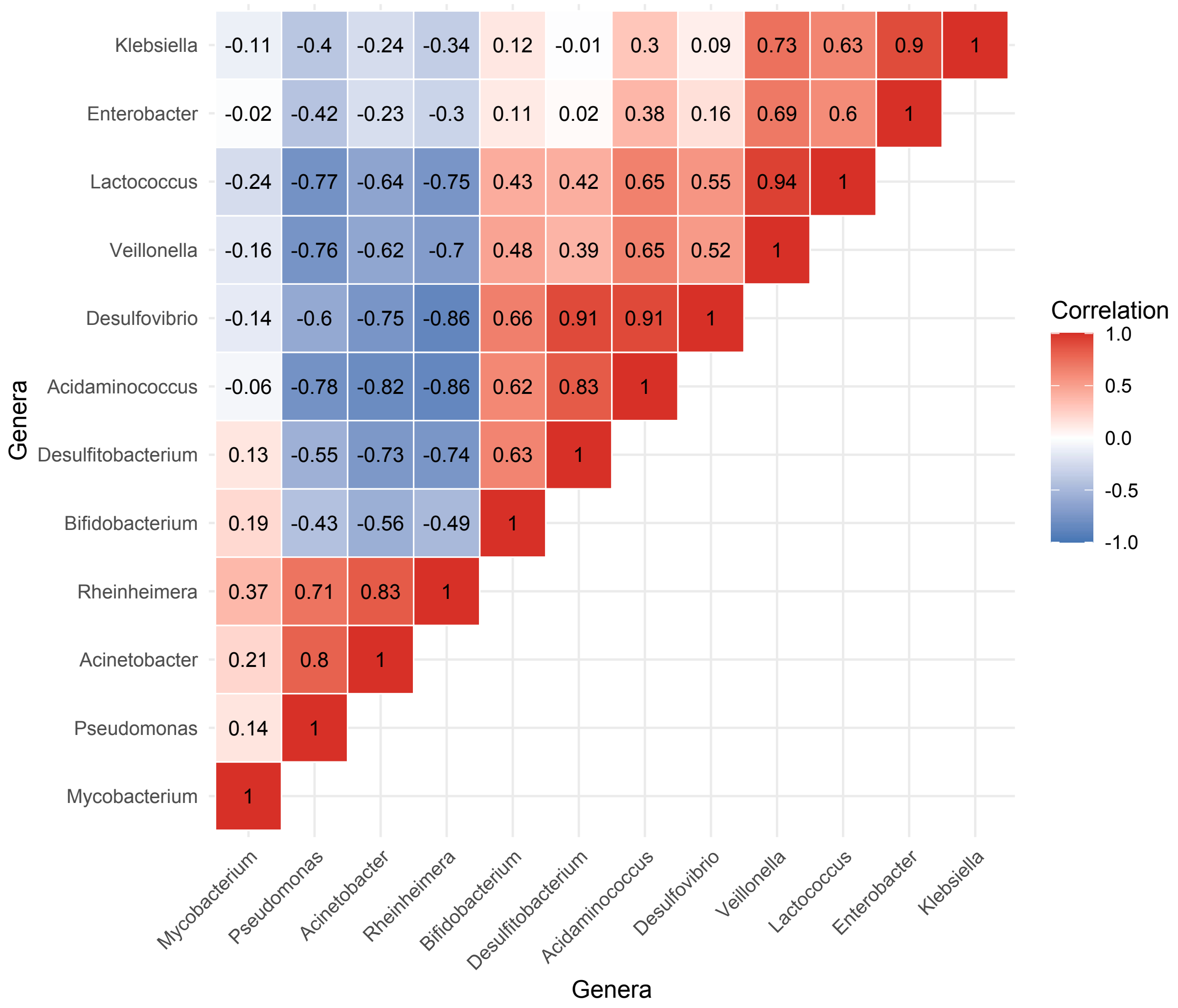

Supplementary Figure S8. Correlation matrix showing the Pearson correlation coefficient between the genera. Only the genera with a high correlation with the ARGs is shown.

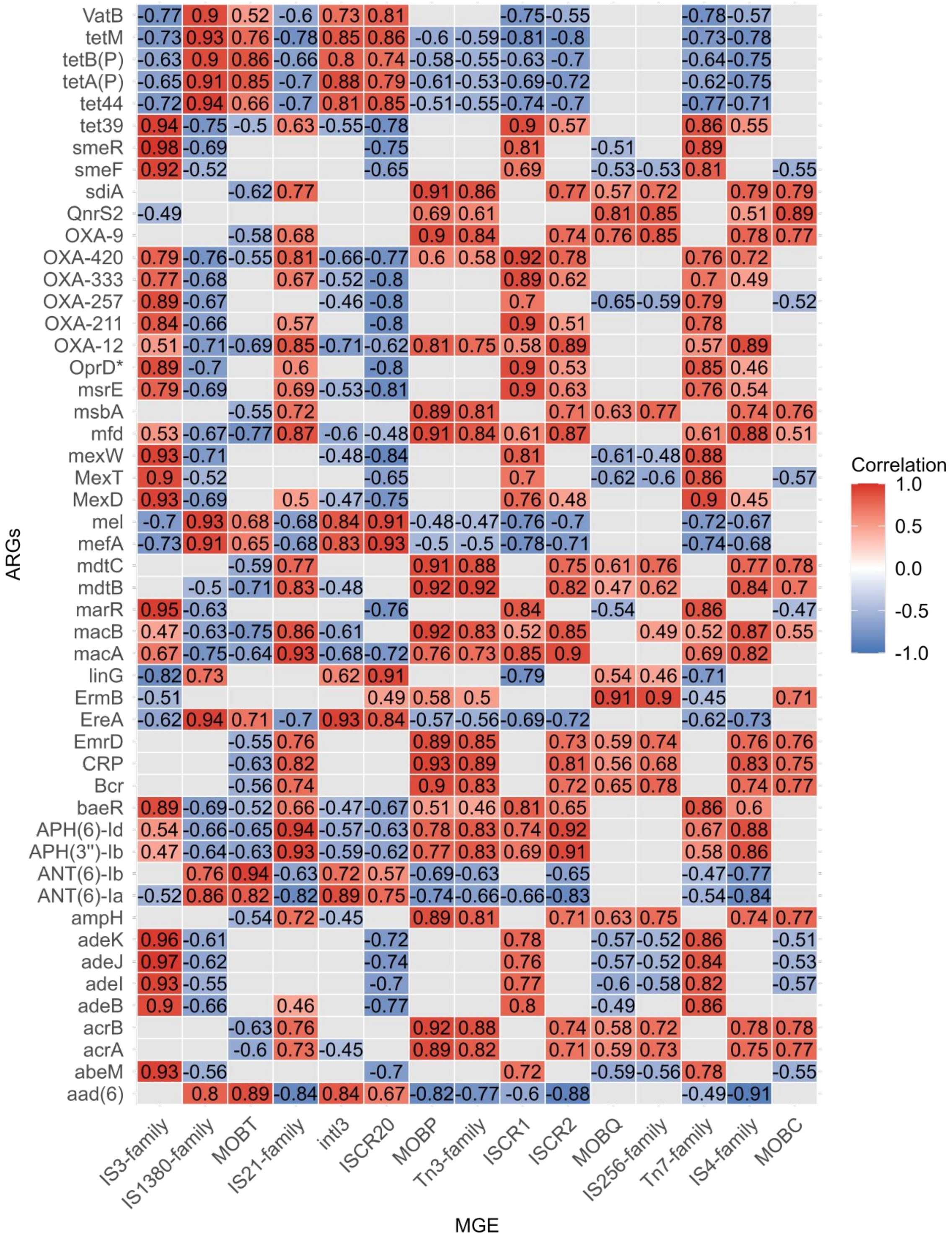

Supplementary Figure S9. Correlation matrix between ARGs and MGEs. The 30 strongest correlations were selected for representation and for the intersections only the significant correlations (after FDR correction) were colored. Numbers within cells correspond to the corresponding Pearson correlation coefficients.

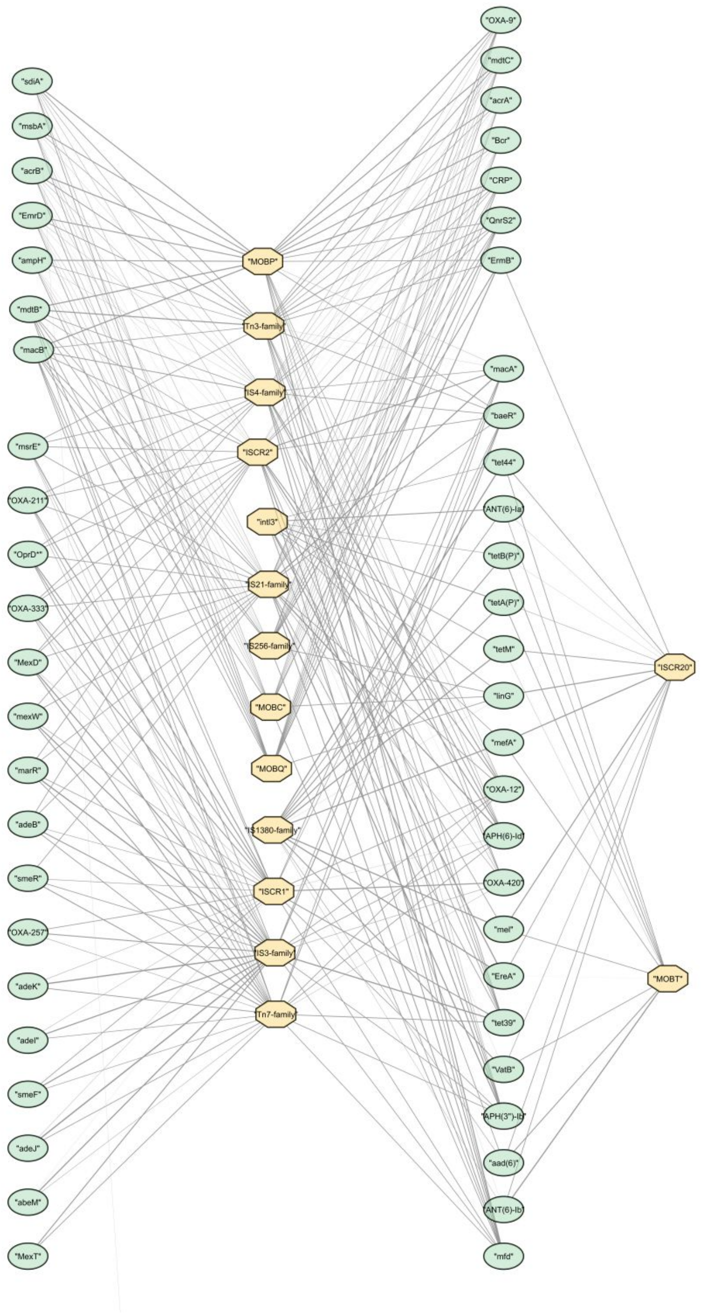

Supplementary Figure S10. Correlation network between ARGs (green circles) and MGE (yellow octagons). Connections represent positive correlations, with an $R_{Pearson}$ > 0.7 and the width indicating the magnitude of the correlation. Asterisks denote gene abbreviations (e.g., *OprD* * stands for *Acinetobacter OprD conferring resistance to imipenem*).
